# Bridging the gap: a qualitative study of providers' perceptions of a partnered crisis follow-up program for suicidal patients post-emergency department discharge

**DOI:** 10.1186/s12888-023-05106-y

**Published:** 2023-11-17

**Authors:** Patricia D. Soderlund, Erick H. Cheung, Madonna P. Cadiz, Hafifa Siddiq, Maria Yerstein, Sae Lee, Kenneth Wells, MarySue V. Heilemann

**Affiliations:** 1 Memory Keepers Medical Discovery Team, University of Minnesota Medical School, 624 East 1St St, Duluth, MN 20155805 USA; 2 Division of General Internal Medicine, David Geffen School of Medicine, University of California Los Angeles, National Clinician Scholars Program, 1100 Glendon Ave., Suite 900, Los Angeles, CA 90024 USA; 3 David Geffen School of Medicine, Department of Psychiatry and Biobehavioral Sciences, University of California, Jane and Terry Semel Institute for Neuroscience and Human Behavior, Resnick Neuropsychiatric Hospital, 757 Westwood Plaza, Los Angeles, CA 90095 USA; 4 Luskin School of Public Affairs, University of California Los Angeles, 337 Charles E Young Dr E, Los Angeles, CA 90095 USA; 5 Charles R. Drew University College of Nursing, 1731 E. 120th St., Los Angeles, CA 90059 USA; 6 Division of General Internal Medicine and Health Services Research, University of California Los Angeles, 1100 Glendon Ave., Suite 900, Los Angeles, CA 90024 USA; 7 Boston University School of Medicine, St. Elizabeth’s Medical Center, 736 Cambridge St, Brighton, MA 02135 USA; 8 Didi Hirsch Mental Health Services, 4760 S. Sepulveda Blvd, Culver City, CA. 90230 USA; 9 Research Center for Health Services and Society, UCLA Jane and Terry Semel Institute for Neuroscience and Human Behavior, 760 Westwood Plaza, Suite 17.369B, Los Angeles, CA 90024 USA; 10 David Geffen School of Medicine, National Clinician Scholars Program, Division of General Internal Medicine and Health Services Research, 1100 Glendon Ave., Suite 900, Los Angeles, CA 90024 USA; 11 Department of Mental Health, Veterans Affairs Greater Los Angeles HealthCare System, 11301 Wilshire Boulevard, Los Angeles, CA 90073 USA; 12 School of Nursing, Factor Building, University of California Los Angeles, Box 6919, Los Angeles, CA 90095 USA; 13 Division of General Internal Medicine, David Geffen School of Medicine, University of California Los Angeles, National Clinician Scholars Program, 1100 Glendon Ave., Suite 900, Los Angeles, CA 90024 USA

**Keywords:** Suicide prevention, Follow-up program, Telephone, Emergency department, Crisis counselors

## Abstract

**Background:**

Effective interventions are needed to address suicide risk following discharge from the hospital emergency department or inpatient setting. Studies that examine follow-up contact methods show promise, but little is known about how follow-up programs are implemented in the real world and who is benefitting. The purpose of this formative evaluation and analysis was to gain insight about the usefulness and value of a partnered suicide prevention follow-up program (academic medical center emergency department partnered with a regional suicide prevention center) from the standpoint of psychiatry resident physicians providing direct care and suicide prevention center crisis counselors making follow-up outreach telephone calls to patients.

**Methods:**

A qualitative thematic analysis was conducted with focus group data from a convenience sample of psychiatry residents who performed consultations in the emergency department setting and counselors at the suicide prevention center crisis follow-up program. Focus group sessions, using semi-structured question guides, were completed at each participant group’s workplace. Grounded theory techniques were used to guide coding and analytic theme development.

**Results:**

Analyses resulted in four overarching themes: valuing the program’s utility and benefit to patients, desiring to understand what happens from emergency department discharge to program follow-up, having uncertainty about which patients would benefit from the program, and brainstorming to improve the referral process. Psychiatry residents appreciated the option of an “active” referral service (one that attempts to actively engage a patient after discharge through outreach), while suicide prevention crisis counselors valued their ability to offer a free and immediate service that had potential for fostering meaningful relationships. Both participant groups desired a better understanding of their partner’s program operations, a uniform and smooth referral process, and awareness of who may or may not benefit from program services.

**Conclusion:**

Results revealed the need for improved communication and implementation, such as expanded inter-agency contacts, consistent provider training, more documentation of the requirements and rules, a consistent message about program logistics for patients, and coordination between the program elements.

## Background

In the United States (U.S.), suicide is a leading cause of death across the lifespan, accounting for nearly 46,000 deaths in 2020 [31]. The National Center for Health Statistics reported a 30% increase in the overall rate of suicide between the years 2000 and 2020 [16]. Of particular concern, individuals (of all ages) with suicidal ideation and/or behaviors were found to be significantly at risk for suicide following discharge from the emergency department (ED) or psychiatric hospital [18, 20, 21, 27]. Based on the American Association of Suicidology & Suicide Prevention Resource Center (SPRC) report, it is estimated that 70% of ED patients never follow through with their out-patient care referrals, and the greatest risk for suicide is within 30 days of leaving the ED or psychiatric hospital [21, 32].

The high prevalence of suicide during this critical period has created a demand for effective interventions following hospital and ED discharge. A variety of interventions, including ED-initiated follow-ups, have been proposed or implemented in limited settings. Follow-up communication has included a variety of methods including phone calls, postcards, in-person, apps, e-mail, and text messaging [7, 8, 10, 23, 26, 35]. Studies generally indicate that repeated outreach services and increased treatment engagement following discharge may help reduce the number of suicide attempts [22, 23, 29]. Qualitative research results informed improvements in the implementation and evaluation in some interventions (such as counseling and restricting youths’ access to firearms and primary care-based suicide intervention), and data from various health professionals were used to identify priorities for suicide prevention [36, 14, 24]. In recent years, the SPRC (the only federally supported resource center devoted to advancing the implementation of the National Strategy for Suicide Prevention, funded by SAMHSA), produced a consensus guide endorsing ED collaboration with their local crisis center to provide “caring contacts” with recently discharged patients [34, 33]. In light of known system-level implementation challenges, further investigation is needed to understand how crisis follow-up interventions are implemented and who benefits from services [2].

**Fig. 1:**
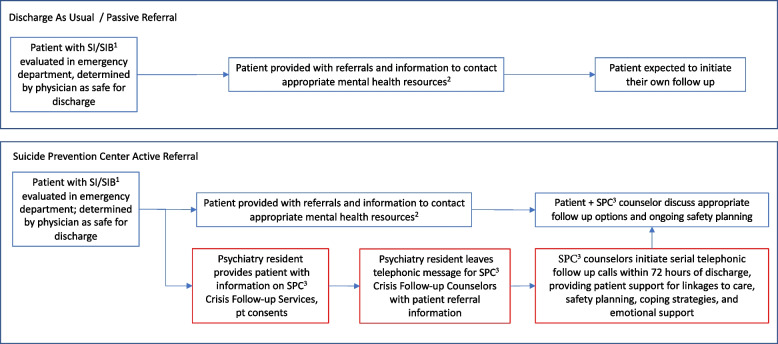
Discharge follow-up protocols 1: “SI/SIB": suicidal ideation or self injurious behavior 2: “Mental health resources”: include but are not limited to psychiatrist, therapist, mental health clinic, partial hospital program, intensive outpatient program, substance use disorder treatment, suicide prevention lifeline, and other community resources for social services, housing or treatment 3: SPC: Suicide Prevention Center

Building on this approach, this article describes a formative, qualitative evaluation of an ongoing suicide prevention partnership that was initiated in 2013. The partnership was formed between an academic hospital ED and an independent regional suicide prevention center (SPC), which established a follow-up program for individuals at high risk for suicide in 2010 through seed funding from the US Department of Health and Human Services Substance Abuse and Mental Health Services Agency (SAMHSA). The goal of the partnership is to provide follow-up services for patients who are at risk for suicidal ideation and/or behaviors during the period following hospital or ED discharge via telephone outreach from counselors at the suicide prevention center’s crisis follow-up program (SPC program). Figure 1 provides an overview of program components. Hospital physicians and social workers refer discharging patients (with their consent) to the SPC program counselors. The SPC counselors then attempt to engage the patient over a series of outreach telephone calls within 24–72 h of discharge, with the aim of providing an array of services: ongoing suicide risk assessment; assistance with safety planning, coping strategies, emotional support; and assistance with establishing appropriate linkage to follow-up care. This type of post-discharge follow-up has been shown to reduce suicidal behaviors and increase engagement in mental health services [17, 29, 30]. By comparison, in the ED “discharge as usual” protocol patients are provided with referral information and are expected to initiate their own follow-up [29, 30, 17].

The inter-agency (ED/Hospital and SPC) nature of this partnership and the referral system is unique in comparison to prior studies. Several prior documented programs have focused on follow-up programs that are owned and operated with the hospital’s own staff. Potential benefits of the inter-agency model include improved patient outcomes due to being connected to specialty trained counselors in the community based SPC program, decreased hospital readmission rates, decreased programmatic costs to hospitals, as well as knowledge to improve scalability to other facilities. Potential risks include errors in communication, lack of continuity of care, and challenges in monitoring data and outcomes.

Evaluation of the partnership began as a quality improvement (QI) project using an inter-agency system framework that combined QI and Exploration, Preparation, Implementation, and Sustainment (EPIS) to inform implementation sciences and partnership of systems [1]. This study reports qualitative results from the mixed methods evaluation of the partnership initiated by a quality improvement team associated with the hospital and ED. The study team conducted a qualitative evaluation using focus group data gathered from healthcare providers, on both sides of the partnership, about the process and effectiveness of the program.

## Methods

### Study design

For this qualitative study, a six-phase thematic analysis [5, 6] using Grounded Theory techniques [9] was conducted with focus group data collected from members of two partnered groups. One group included Psychiatry Resident Physicians (psychiatry residents) who worked in the EDs managing direct patient care, including discharge aftercare. The other included Suicide Prevention Center Counselors (counselors) who received specialized training to participate as a staff member in the Extended Crisis Follow-Up program (EFU program). Approval for this study was obtained by the university’s Institutional Review Board (IRB#19–001165), and all participants in this study gave written informed consent. Focus group questions centered on the usefulness and value of the suicide EFU program from the perspective of both the psychiatry residents and the SPC counselors. Open-ended prompts were used to address, at a minimum, the following domains of inquiry:

- 1) Whether or not services addressed specific population needs,
- 2) How the inter-agency suicide prevention follow-up program was implemented and suggestions for improvement,
- 3) Exploration of program operations to inform formative program evaluation and suggestions for modifications.

### Sampling and recruitment procedures

Purposeful sampling was used to recruit participants for parallel focus group sessions including one series of focus groups for psychiatry residents and another series for SPC counselors. Psychiatry residents were eligible to participate if they were working at the academic hospital ED between 2017 and 2019 (recruitment ended October 2019), had been working there for at least 6 months, and had managed at least 15 ED patients with suicidal ideation and/or behaviors. The SPC counselors were eligible to participate if they worked in the follow-up program for at least 6 months between 2017 and 2020 (recruitment ended February 2020) and had participated in at least three ED follow-up cases.

### Data collection

A semi-structured question guide was used in focus group sessions that were conducted at each participant group’s workplace. A total of 5 focus group sessions were completed (2 sessions with psychiatric residents, and 3 sessions with SPC counselors). One of the 5 focus group sessions included 3 SPC counselors and it extended to a second session, however, two new participants joined for the second session. A total of 18 participants participated overall in the focus group sessions. Each session was digitally recorded and lasted approximately 60 min.

The semi-structured question guide included open-ended questions that were designed to be exploratory to gain each group’s perceptions about the value of services offered, understanding of and experience with running the program, and how program operations and services could be improved. At the completion of each session, individual participants received a US $25 gift card. All digital recordings were uploaded to the university-approved, secure, HIPAA-compliant cloud-based storage service. Audio files were transcribed verbatim by a professional and secure transcription service. The team verified accuracy and de-identified transcripts in preparation for analysis.

### Data analysis

Thematic analysis was employed in 6 phases (data familiarization, initial coding, clustering of codes, initial themes defined, theme refinement, and writing up findings). First, the analysis team familiarized themselves with the data by reading and re-reading the transcripts while making notes about analytic ideas for potential themes. Line numbers were used on transcripts to heighten organization of data and notes. In phase 2, every transcript was coded line-by-line [5, 6, 9] by 1 researcher. Subsequently, each coded transcript was reviewed by at least 1 other researcher who added to the coding when appropriate to identify and label action or other details not already coded by the initial researcher [5, 6]. Memos were written to identify early hunches and aspects of themes identified during the coding process. Coding was guided by the analytic approach described by Saldaña [28], using strategies that included process coding (using gerunds to focus on the actions of participants in the data), emotions coding (to highlight feelings expressed by participants in the data), and values-based coding (to underscore data that indicated values held by the participant) [28]. While coding, researchers made analytic notes about potential themes based on the active process of coding in the transcript margin to preserve the location of the actual data. In phase 3, codes that showed broader, shared meaning across transcripts were clustered together. Examination of these clusters through comparison of data with data and codes with codes [9, 11] allowed data to be classified into initial themes. In phase 4, initial themes were defined and reviewed for patterns across the entire data set. A chart of all themes allowed for scrutiny so some themes could be combined while others that were thin were discarded. In phase 5, the remaining themes were refined, clarified, and named. Phase six included writing up the findings [5, 6].

## Results

### Sample

A total of 9 psychiatry residents and 9 SPC counselors consented and participated in the study. Psychiatry residents were between the ages of 28 and 35 years of age, and SPC counselors were between the ages of 24 and 59 years of age. All focus group sessions included male and female participants (Table 1).

**Table 1:** Demographics

	**n**	**Proportion (%)**	**M**	**SD**	**Median**	**Minimum**	**Maximum**
Age (years)							
ED Psych Residents	9		30.89	2.26	30	28	35
SPC Counselors	9		37.89	11.54	38	24	59
Female							
ED Psych Residents	6	66.6					
SPC Counselors	5	55.5					
Work Length (months)							
ED Psych Residents	9		26	10.01	27	15	40
SPC Counselors	9		15.78	9.77	14	6	36

### Analytic overview

This analysis resulted in 4 themes: valuing the program because it is useful and benefits patients, having a mutual desire to understand what happens from the ED to discharge to program follow-up, uncertainty about which ED patients would benefit from program services, and brainstorming to improve the referral process. See the main themes and subthemes outlined in Table 2.

**Table 2:** Main themes and subthemes that emerged from provider focus group sessions

**Main theme 1: Valuing the program because it is useful and benefits patients**	
Subtheme 1 (Psychiatric Residents):	Subtheme 1 (SPC Counselors):
Valuing the program because of their belief in the potential effectiveness: “*An active referral that’s actually going to work*”	Valuing the program because it allows them to be a safe “*bridge*” for patients
**Main theme 2: Having a mutual desire to understand what happens from the ED to discharge to program follow-up**	
Subtheme 2 (Psychiatric Residents):	Subtheme 2 (SPC Counselors):
More than checking off a “*checklist*”: Desiring to know “*what actually happens in the program*” after a referral is made	Fixing the disconnect: Desiring to know “*what’s happening on the side of the ED?*”
**Main theme 3: Uncertainty about which ED Patients would benefit from program services**	
Subtheme 3 (Psychiatric Residents):	Subtheme 3 (SPC Counselors):
Struggling to know which patients are “*good fits*” for the program	Wrestling with uneven realities: “*first timers*” and those with “*many diagnoses*” can potentially both benefit
**Main them 4: Brainstorming to improve the referral process**	
Subtheme 4 (Psychiatric Residents):	Subtheme 4 (SPC Counselors):
Envisioning a more streamlined, efficient referral process	Having a wish list that includes getting key contextual and accurate referral information

### THEME #1: Valuing the program because it is useful and benefits patients

### Psychiatry residents valued the program because of their belief in the potential effectiveness: *“An active referral that’s actually going to work”*

Psychiatry residents optimistically regarded the EFU program as an *“active”* referral service meaning it was provider initiated instead of patient initiated (refer to Fig. 1). Their appreciation for the program stemmed from a belief that if clinicians take the first step, post-discharge follow-up would be more likely to occur than if it was left to the patient. For example, the traditional discharge resources would typically include a set of county agency and private provider contacts that the patient would be advised to call. The program seemed to be actionable specifically because it was designed so post-discharge follow-up would occur by phone with clients when at their place of residence over an extended period. Since feedback about clients’ use of referral services had never before been available, psychiatry residents speculated that patients were more likely to follow-through since it was an active referral to a real program. One resident stated:

*Most people you’re sending out into the wild, you know. It’s an active referral that’s actually going to get back to them. Whereas like those community referrals that we give them are just, you know, it’s a low probability that that’s going to work out. But this actually happens, it’s really you know, it’s kind of cool.*

One psychiatry resident referred to the efficacy of an extended follow-up research study on clients with suicidal ideation and how that study showed promise. He emphasized the importance of outreach and stated, “*They were able to show a reduction in … attempts and ideation in this group that had the most robust outreach after visiting an ED for suicidal ideation*”. Reflecting on that research study prompted the resident to consider the ease of the process of referrals to the EFU program services, and how all eligible clients could benefit, even those with established psychiatric care:

*I usually still offer it [for clients with established mental health care] because that’s still, like, for them to go to the therapist, relies on them to some degree, that they have to put in more effort, whereas [receiving] the call is a very minimal effort on their part.*

EFU program services were also viewed as a safeguard when discharge options were suboptimal. One psychiatry resident participant explained, *“In the absence of some other enriched discharge plan or support program this is a safety net in a way.”* Another resident described it as an additional layer of emotional support:

*Maybe promote kind-of a safety approach in showing the patient that there’s more support there for them, in this time of need. I think that could have some proven benefit, if that’s the case, even if it’s just a phone call.*

### SPC Counselors valued the program because it allows them to be a safe “*bridge*” for patients

SPC counselors highlighted the ability to develop a meaningful relationship with patients over an extended period of time as a distinct and valuable aspect of the EFU program. They appreciated the unique opportunity to learn more about participants’ life histories and be with them long enough to bear witness to participants’ progress, as well as potential regressions in terms of their treatment.

*I think the ability to build a connection for—whether it be six weeks, eight weeks, which we don't really get, you know, on the other side of the crisis line. To be able to build those connections and kind of hear someone's story—whether it be we hear the progress or a decline in some cases—being able to literally follow up and hear those stories and be that support system for those weeks is very different than what we normally do.*

Another SPC counselor recalled a time when they were able to follow a patient’s initial and ongoing reactions to their medication regimen. They recalled the patient sharing that the medication was initially efficacious, but later on stopped working and led to their hospitalization. Commenting on the value of an ongoing relationship with this patient, they stated, *“… that's the kind of insight that you get when you talk to the people for prolonged periods of time, you know?”* Similarly, another SPC counselor described a patient who willingly admitted to having continued thoughts of suicidality; the counselor then tied this to feedback regarding the EFU program.

*I was that person who she felt safe with, to be able to admit like, "Hey, things aren't better. Just because I went to the hospital, and I've been doing this program for two weeks doesn't mean I'm suddenly better." So, I think a big goal is to be that space for people to admit that maybe it's not going to always be perfect every time.*

SPC counselors identified some key factors that allowed them to create a safe space for patients. They pointed out the fact that EFU program services were *“free”* and *“immediate”* which made them much more convenient and accessible than other types of therapeutic services.

*I think that because we're free, it's a free service, I think it really helps people to kind of plug into—you know, be a part of it. And then also that it's so immediate because we call within 24 hours of getting the call from the doctor. And that it's almost like...it's not like they're seeking a therapist and it takes a couple weeks or several days to find someone. It's sort of like…, there's someone there calling me, wanting to talk.*

SPC Counselors also noted the benefits of the EFU program being a *“bridge”* for patients between discharge from the hospital and beginning formal mental health therapy. Thus, it functioned like a *transition* service. They saw it as a crucial period for maintaining engagement with patients.

*The people I've dealt with seem to appreciate the extra support while they're waiting to get into some kind of ongoing help. They might have to wait a week or two for an appointment and then we can speak to them during that time and offer support. I feel like that's important.*

Multiple SPC counselors highlighted the importance of *anonymity* in allowing patients to confide in counselors more honestly compared to mental health therapists or other service providers.

*I think that they still feel a little bit anonymous, where maybe sometimes it's hard to—even when you have a therapist, you know, you're in the same room with that person, you're looking at that person in the face. So maybe you don't want to say, "I'm homicidal," … in fear of “I'm going to get arrested” or “I'm going to get put back in the hospital,” where that's not really what they want or need. They're just – they want to express themselves. And I mean, obviously they're expressing themselves, they're trying to keep themselves from intrusive thoughts, right? So, I think that the whole – like the phone conversation gives them that anonymous feeling. Like we don't know what they look like, we don't have any judgment, it's judgment-free. And they don't know what we look like, either, because it works both ways, right?*

SPC counselors sensed that patients appreciated the lack of judgment coming from fellow counselors overall, noting that patients also had less fear. They saw this as being unlike the approach typically taken by therapists when working with suicidal patients.

### THEME #2: Having a mutual desire to understand what happens from the ED to discharge to program follow-up

### More than performing a *“checklist”:* Psychiatry residents desired to know *“what actually happens in the program”* after a referral is made

All psychiatry residents in our sample reported using follow-up services for their ED patients. While enthusiastic about the idea of a linkage service, residents lacked knowledge and understanding about the program’s fundamental features. One responded with an optimistic but speculative response:

*One of the nice things is supposedly the people who call the patient will help to make sure they’re linked to services, so it’s more than just like, “Hey, are you okay?” It’s like, “Hey, like, do you have a follow-up appointment?” And, like, apparently some help linking people…”*

Psychiatry residents lacked information about the operations of the EFU program and its overall effectiveness or outcomes. Consequently, residents varied in their readiness to make a referral and the actual number of referrals they made to the program. One resident had been referring all discharged clients who were *“at risk for persistent suicidal ideation.”* He concluded that the program could only benefit patients because, by nature, it was a minimal risk follow-up telephonic program. He could not see any potential harms:

*The risk is essentially zero. That’s like, I hear if you get that phone call and you’re not interested or you don’t take it, like, that’s it. That’s the downside. I mean, it’s a waste of that person’s time who reached out, but I would actually argue that it’s still valuable.*

Yet, other residents voiced doubts about the potential benefits of the program. One resident stated, “*If I had knowledge that these phone calls were effective and that patients were actually picking up, then I probably would be referring more”.*

All psychiatry residents in our sample emphasized the need for a more in-depth understanding of the quality and extent of the services rendered by the program. They lacked knowledge about the quality and extent of services offered and desired to learn more. One resident posed the following questions:

*I would want to know how long that they stayed in the program or how many phone calls they had afterward. Like, if there was any change in their suicidal thoughts, and if they’d started any sort of mental health treatment, [that] would be interesting to know, too.*

Another psychiatry resident recommended that a SPC counselor should lead an in-service for new incoming residents. He wished he had received an in-service session early on that would have included first-hand information about case accounts along with a description of program operations. He believed such an in-service could positively impact residents’ attitudes and improve their practice of referring patients into the program.

*I think it would be nice if they came during our-like intern orientation or any point someone from [Community Mental Health Center] and explain like, ‘Hey, this is what we do on the backside when you make this referral’, so we have a better understanding of that. “Here’s some vignettes of patients and experiences we’ve had, and these are the outcomes we saw. Thank you for making that referral because you provided a benefit in X, Y, and Z ways”. I think it would be great and doing that early on in the residency rather than later just to change our kind-of frame of mind when we are making this consult, rather than it just being part of our checklist.*

### Fixing the disconnect: SPC counselors desired to know “*what’s happening on the side of the ED*?”

Several SPC counselors reported a lack of clarity regarding how the program worked for the psychiatry residents on the ED end. SPC counselors who did have some understanding of the ED’s services relied on their own previous experiences with the program or with emergency medical services in general. For instance, in response to a question related to ED services, 1 program counselor stated, *“I did [know] only from life experience and other jobs. But I don't know that they specifically trained me at what goes on, on [the ED] side of things.”* Similarly, another SPC counselor indicated it was only after working in the EFU program for a while that they learned the basics of what ED physicians do:

*I think, at least for me, knowing what's happening on the side of the ED. Because we're told this is what could be happening. This is what you're supposed to—someone’s going to the ER and this is maybe what's happening. But we don't really know necessarily what the ED is doing. I think now I know because I've been here long enough to figure out that a resident or someone gives this person a consent and asks them, “Do you want to participate?”*

SPC Counselors believed the primary role of ED psychiatrists was to inform patients of the EFU program and obtain consent. Thus, SPC counselors expected psychiatry residents to provide clear, detailed information about the program to patients and instruct them to expect a call from a SPC counselor. They hoped such sharing of instructions would reduce the confusion counselors sometimes encountered when they called patients for the first time:

*I've been told that in theory they're supposed – the doctors are supposed to explain the program to them. And then someone would call, we're supposed to assume they've already given consent to be called and that, so it’s okay to leave a voicemail and that kind of thing. But yes, sometimes, half the time they're like, “Oh, yeah. Hi.” And then the other half they're like, “What? Who? What?” they have no idea what it is.*

SPC counselors estimated that only about *“fifty-fifty”* or *“half the time”* their patients reported their psychiatry residents informed them of the program. They appreciated when the psychiatry residents did inform patients about the program but postulated that psychiatry residents needed to be more consistent in adequately preparing patients for the program. One SPC counselor suggested that increased monitoring would help improve the ED side of the program.

*I think, if you wanted to track that, you would need to add the protocol question when we talk to them: Did the hospital explain to you the program? And then we would check yes or no. And then you would follow up tracking it, would be the only scientific way to go about it.*

Finally, 1 SPC counselor expressed the potential benefits of conducting an in-service training so that psychiatry residents and counselors could meet each other and get to know each other’s roles in the program.

*So, if there is a way we could meet some of these residents or some of the people that are in charge. And just, here at our organization we're very big on doing like in-services and learning our partnerships. So, we do things with two-on-one, we do things with county child and family services, in order for our counselors to be able to see who they're working with or who they're referring to. So, in the same manner, I would want our follow-up team to have that experience of going to go see the ED and meet some of these doctors or residents. And they, too, to meet us so that they also feel more comfortable …*

### THEME #3: Uncertainty about which ED patients would benefit from program services

### Psychiatry residents struggling to know which patients are *“good fits”* for the program

Although the program was designed to help patients, psychiatry residents lacked clarity about the type of patients who would and would not benefit from follow-up services. Opinions about who should be referred were speculatory and based on psychiatric diagnoses, personality traits, social support status, and presence of long-term psychiatric care. One resident claimed that all eligible participants were potentially worthy candidates,

*I feel like the only people who I would think aren’t good fits is if they’re not consenting or if they don’t have a phone number that they can be contacted by….*

Other psychiatry residents narrowed it down to more specific inclusion and exclusion criteria based on their assumptions. One resident described a model candidate saying:

*I think an ideal patient for this is a lonely patient, an isolated patient, somebody that really benefits from conversation and outreach. The more robust their social network, the less I would—maybe the less they need this*.

When considering who to exclude, psychiatry residents relied on contextual factors and considered them across different patient population groups. One resident regarded positive “caretaker status” as a reasonable exclusion criterion for the EFU program, saying, “*Cognitively impaired with caretakers’ kind of thing. We see autistic people who want to kill themselves. I don’t know. I doubt I’d refer them.”* This resident raised other concerns that made criteria of who to refer less clear. They pointed out the ramifications of referring a suicidal patient with a personality disorder, saying:

*Much of the time in borderline personality disorder those are like—you know, they’re instrumental, right? They’re saying, "I’m suicidal," or gesturing or whatever it is they’re doing in order to get something else. Giving some patients the opportunity to escalate on the phone could be problematic. Part of it might just be my like counter-transference and I don’t want to subject the person [SPC counselor] to that.*

To determine appropriate candidates for the program, psychiatry residents recognized a more granular need to understand the EFU program’s goals. One resident revealed confusion about the motives of the clinic sponsoring the program. He reported:

*I also wonder what [name of mental health clinic]’s goal is in this process. I mean, certainly, it’s a nice, shared goal to have improved triaging services and step down or acute crisis management, sub-acute crisis management. But if their goal is to transition from phone calls to outpatient services, this is a referral mechanism for [name of mental health clinic] to have more patients in care, maybe they’re okay with dysregulated character pathology. And certainly, that phone call might be a little...rich. But maybe—I don’t know, you know, maybe it’s still an access point.*

Even though child and adolescent patients were eligible to participate in the follow-up program, when asked about the utility of referring younger populations, psychiatry residents were admittedly unclear about the program’s age requirement. One resident said, *“Well, now I don’t remember the age cutoff for the program, does anyone know?”* Another responded with a question saying, *“Sixteen maybe, I’m not sure, or twelve, I don’t know?”, and a third added, “I actually thought it was only adults”*. For 1 resident participant, the focus group discussion resulted in doubts about their own referral practices for adolescent patients. They reassessed their own actions, saying: *“Now I’m not sure if that was appropriate”*.

When considering the referral of younger patients into the program, residents established the circumstances of younger populations as fundamentally different saying, *“they’re never isolated”* and when discharged home, *“there’s always a caregiver”*. These considerations ultimately redefined their perceptions of the referral process. One resident posed questions that revealed the complexity of making a referral from their point of view:

*Or is it helpful to think about the patient with his or her family as the unit of referral? I mean, assuming there’s still some driver of chaos or destabilization in their life that may be leading to suicidality or interpersonal problems. If I’m thinking about this referral also as like a temporizing measure to get to an appoint—you know, you have an appointment coming up in a month, what are we going to do?*

Despite the questions of who should be referred, there were some success stories. One resident was optimistic about their experience with a *“family referral”*. Nonetheless, they expressed lack of certainty:

*And they [parents] were really onboard with the follow-up services, and were really adamant that, “You need to answer this phone call when it comes to you." And they were really encouraged to have something there. So, I don’t know. It seemed like a positive thing to be able to give them.*

### SPC counselors wrestling with uneven realities: *“First timers”* and those with *“many diagnoses”* can potentially both benefit

SPC counselors expressed varied opinions regarding the type of person who would benefit most or least from program services. However, most agreed that patients who were self-motivated and eager were much more likely to benefit from services.

*I was blessed with the ones that they were really willing to help themselves and they were eager. And so this program was wonderful for them. And it worked and they were so happy and we really had a good bond. And it was like the last call was like a lot of crying and, you know. So, it was a very good, good, useful program for them.*

SPC counselors identified a few specific factors that may make it more likely for patients to express such eagerness. First, patients with little to no previous experience with hospitalization, therapy, or other types of treatment related to suicidality tend to approach the program more openly and with excitement.

*But I mean it's really exciting when you get someone that's never done a safety plan, that looks at that safety plan and is all excited about identifying triggers and coping skills. I mean, it's just there's a lot of energy for that when it's brand-new for somebody, really, really helpful, a tool that they'll use …*

SPC counselors reported that the services they offered were almost like a revelation to these patients, a solution that they had never considered or been presented with before. Thus, the counselor thought they may be more eager and motivated to try something new to help them with their suicidality. Similarly, several counselors indicated that young patients seem to benefit more from program services.

*Yeah, I definitely feel like it's usually the … first attempt or first hospitalization and they're usually younger and just have a little more to look forward to in life. I don’t know, at least that's what I've gathered just from like certain EFU clients that if you go through the whole program with them, by the end they're like—you know, they have a new job or they're starting fresh on a lot of things and really taking it seriously. Because it's scary for them, because they're really young and it's affected their family. They're still connected with all these people in their community, so there's a lot hanging in the balance. And I think that's why they take it more seriously.*

Like those who have minimal experiences with hospitalization or treatment, SPC counselors found that younger patients were more compliant with the program, or at least motivated to try EFU services; this allowed them to see positive changes in their lives and attribute the improvements to their engagement in the program. In contrast, SPC counselors saw patients who were older, those with more complex mental health diagnoses or histories, or those with more experience with hospitalizations or treatment as less likely to benefit from their services.

*Other people have been in and out of the hospital so many times that it's—you know, when you talk about coping skills, "Oh, I know all that. I've done all that. I have a million safety plans." So it feels less helpful maybe, maybe even a little more hopeless for me as a counselor. Like what can I even offer this person? They have 10 different diagnoses and they've been to 20 different therapists in their life, so it can be challenging.*

Patients who had been through multiple hospitalizations or treatment experiences, according to SPC counselors, already knew the interventions presented by the EFU program. Although safety planning or other services may have worked for them in the past, there was nothing new or revelatory that they felt they could offer at this point, thus leaving the counselors feeling generally defeated and unable to help these particular patients. However, these characterizations were not absolute, as exemplified by a SPC counselor who described a patient with multiple diagnoses and a complex history who ultimately benefited from the program.

*… now, the person that popped in my mind when you asked that was someone that was not a first-timer, that was a 40-something woman with many diagnoses and self-harm and struggling in and out of therapy. Most of our calls were just about the self-harm issue and just baby steps, like she would keep her sharps in her nightstand and pills. She would overdose, like overmedicate. So, I got her to put them in her closet in a bag. So little baby steps that were really hard for her but she did it.*

This SPC counselor felt they made a difference by gaining a deep understanding of what the patient needed and taking the “*baby steps*” necessary for the patient to make progress. Thus, for this SPC counselor, it seemed that the effectiveness of the program depended largely on their ability to learn about each patient’s specific needs and tailoring services to those needs.

### THEME #4 Brainstorming to improve the referral process

### Psychiatry residents envisioned a more streamlined, efficient referral process

Psychiatry residents described the EFU referral process as potentially burdensome and emphasized the work of “*another step*” in a busy ED environment. One resident explained:

*It’s just another step, another phone call to make in the evening when you may have already tried to call a therapist, call a family member, call someone else and when you’re trying to wrap up the rest of the work, and this is one more step.*

Another resident commiserated saying,

*So, yeah, so it usually means that you have to go talk to the patient again, which sometimes it’s a significant amount of work when we’re really busy if we have a lot of patients backing up.*

Nonetheless, psychiatry residents discussed strategies and welcomed ideas that could potentially help streamline the process. While considering the delegation of referral activities, a resident expressed uncertainty about who could initiate the referral saying, *“I'm wondering, I don’t know when I was told about this service, if I was ever clearly instructed that it had to be an M.D.”* One resident contemplated this saying, *“they make us think we have to do that”* and “*I don’t know, maybe there’s a good reason?”* While some residents wondered why the referral process had not been delegated to other health professionals, others were encouraged by the idea of a practical interdisciplinary process that might involve *“the nurse that’s sitting right there next to them [patient]”* and “social workers”.

Psychiatry residents also believed that technology could be used to help leverage the EFU referral process. They engaged in problem solving in the focus group, seeking a way to integrate EFU referrals into the electronic health record (EHR) system so it would be smooth and time saving. One resident suggested:

*… just put it in [the EHR system]. I mean, I’m happy to sign it when I put it in. I don’t think it’s about shirking work. It’s about making it more efficient. If you did that, I’m sure they [EFU program] would see the volume of consults go up too, if that’s what they want.*

Another resident agreed saying:

*A hundred percent. It should be an order in [EHR System]. If that were a possibility, that’s like an obvious improvement to this. Because I remember recently the [EFU program] number changed, and then you're just like...so if you could search your email—I mean, we are slammed busy in this ED. This is not something you want to be wasting time on, and it’d be really nice if it was part of the discharge order set or something.*

### SPC counselors’ wish list includes getting key contextual and accurate referral information

SPC counselors indicated their desire for more comprehensive contact information from psychiatry residents so they could discuss pertinent questions regarding their patients. One counselor offered a potential solution in having a list of ED doctors’ names and callback numbers available at the call center:

*I don't know, if we had a list here we could at least put it together and have the right spelling and have the right contact number. Because we often don't get callback numbers, either. So, if we had questions for the doctor about the patient, we don't know how to get in touch with them. I mean, we know they're with the hospital, but we don't—it would make it easier if we had a number.*

Relatedly, several SPC counselors lamented a lack of uniformity in the amount or types of patient information physicians left in their voicemail messages. They discussed a need for vital patient information, including *“the basics like name, age”* and *“discharge time”*. Further, they wanted to hear about patients’ relevant psychological history. One emphasized the practical value of this information saying, *“… it would help to know if the person has been previously diagnosed with any kind of mental disorder and if they're receiving treatment for that, because that gears our conversation.”* Another wished they could know a physicians’ appraisal of how receptive a patient might be to the EFU program:

*I mean, I would also argue the presentation of the person, of the patient. Because if we know, okay, patient is hesitant towards program, we may approach how we speak to them for the first time differently versus someone who's eager to—even that little tidbit of information would be able to gear our first contact and how our voice tone … is, how many times potentially we go ahead and try to make contact, as well.*

Finally, SPC counselors emphasized the importance of being informed if the patient actually consented to the referral. They wished referring psychiatry residents would give more context when they provided alternate contact numbers for a patient. Counselors admitted it was at times difficult to navigate conversations and maintain confidentiality when they were unable to speak directly with the patient on a follow-up call.

## Discussion

This qualitative exploration describes how healthcare providers perceived the process and effectiveness of a real-world suicide prevention telephone follow-up program that was formed jointly between an academic ED and a regional suicide prevention center. The 4 key themes that emerged indicate ED psychiatry residents and SPC counselors regarded the EFU program as valuable, desired to understand how the “other” side of the program operates, felt uncertain about which ED patients would benefit, and embraced ideas for improving the referral process. Within each theme, findings uncovered the nuanced experiences and perspectives of both the psychiatry residents making referrals to the EFU program and the SPC counselors making the outreach follow-up calls to patients. Application of the EPIS framework to this analysis highlighted that while there was enthusiasm for the EFU program, there was a need for improving communication, collaboration, and system effectiveness within and between agencies to inform the sustainment phase of the process [1]. For example, the EPIS framework emphasizes “inner and outer” context (within and across systems) and acknowledges the diversity of patient/family experience and context, which suggests tailoring communication for different age/cultural groups as well as promoting system collaboration to implement the EFU program [1]. Our emphasis on partnership development and communication is consistent with a “partnered” or “engaged” approach to implementation [19].

The SPRC produced a consensus guide for emergency departments in caring for adult patients with suicide risk, which included a recommendation that EDs “consider establishing an agreement with a local crisis center that allows its staff to make caring contacts with recently discharged patients” [4, 25, 33]. These contacts are intended to facilitate adherence to the discharge plan and promote a feeling of connectedness by demonstrating continued interest in the patient [3, 17, 13]. However, according to the 988 Suicide and Crisis Lifeline (formally known as National Suicide Prevention Lifeline) website, only 38% of local Lifeline centers in the US have formal relationships with one or more EDs [15]. It has been anecdotally reported that some hospitals found that contracting with a crisis center, such as a 988 Suicide and Crisis Lifeline local center, has been effective in supporting patients through follow-up calls, re-assessing suicide risk, and maintaining a connection until patients are seen in outpatient care; however, there are few studies on these partnerships [25]. Systems for extending caring contacts require scrutiny and ongoing quality improvement to assure that the people in need are effectively connected with appropriate follow-up care [12]. Since there may be significant costs associated with employing personnel to engage in such outreach to patients, funding for programs that foster authentic, appropriate, and effective connections is crucial to develop and sustain such services [12].

Our findings suggest that partnerships between EDs and suicide prevention centers that specialize in suicide risk screening, assessment, triage, and management, may potentially benefit patients, communities, and health systems if efforts are invested to assure that the system collaboration is patent. Psychiatry residents valued the EFU program since it was an active referral, having an advantage over standard of care passive referrals that require patients to initiate follow-up. This feature could be crucial for high-risk patients who may not have the capacity or desire to make contact. SPC counselors embraced the role of being a transitional facilitator and saw themselves as well suited to their role, viewing their anonymity as an advantage. Phone, versus in-person services, helped counselors feel they could build trust within a judgment-free space, as well as foster honest interactions. The insights provided by psychiatry residents and SPC counselors indicate features of the EFU program that may be key to its efficacy.

While interventions are needed to address suicide risk following discharge from an ED or hospital setting, our qualitative findings indicate that gaps in communication between partners may have negative consequences for providers and patients. Psychiatry residents considered the EFU program to be a “safety net” that may greatly benefit patients at risk for suicide after discharge. However, fluid communication between partners, timely referral, and reliable follow-up is needed to help those most in need of follow-up. SPC counselors in this study reported challenges with the enrollment process, stating some patients failed to understand what the EFU program was, making their extended follow-up services feel more like a “cold call”. It is not immediately clear whether this communication gap is due to hand off between agencies (via a recorded phone message), and whether this problem would persist even in an in-house program operated by the hospital/ED. Efforts to determine how to fortify the existing system or eradicate this communication gap may benefit from additional studies focused on patient perspectives on the flow from the ED to the EFU.

The findings of this study suggest that EFU providers may question the worthiness or utility of the EFU program for different types of patients, as opposed to simply referring all patients who meet the criteria regardless of diagnosis or condition. In particular, psychiatry residents questioned the motives (whether conscious or unconscious) of patients with borderline personality disorder who may articulate suicidal ideation as instrumental or for secondary gain (such as emotional reward, attention from others). While they voiced awareness of their own countertransference, they simultaneously expressed concern about the re-enactment of patients’ transference, attention-seeking, or maladaptive behavioral patterns with the SPC counselors. Similarly, counselors suggested a nuanced view of some patients potentially benefitting more than others. For example, they suggested that patients range from those with no previous experience creating a safety plan, to those who are chronically suicidal, and that interest in creating a meaningful safety plan and/or the program may be higher for patients with new-onset suicidality but lower for those who are chronically suicidal or chronically relapsing patients (whether associated with borderline personality disorder or not). Thus, the recognition and understanding of the needs and desires of the range of suicidal patients who may present to the ED, warrants not only the attention of clinicians, but from researchers too. More evidence-based insight is needed on whether it is important to differentiate within suicidal populations, and whether specialized approaches, training, or support systems may be useful within the 988 Lifeline system [15].

When psychiatry consultants in busy and often overcrowded EDs have an established relationship with a patient, curating a referral to an outside system can be time consuming for providers and patients/families. Streamlining procedures to allow follow-up referrals by interdisciplinary care providers, and leveraging technology such as websites, may have potential to reduce barriers and expedite referrals. Training providers on ED teams, such as social workers and nurses, could help foster a more seamless referral process across the inter-agency partnership. Furthermore, embedding follow-up referral activities within an existing and well-established ED electronic health record (EHR) system increases likelihood of implementation, documentation and potential success [1]. This was identified by residents as a potentially high yield way to reduce work burden and ensure fidelity of the program. This standardization of referral activities within the EHR system could also improve trust and communications across systems, serving as a signal of reassurance that consistent and adequate referral practices are followed [1].

On the other hand, systems of automatic referral that foster efficiency may have the downside of hampering the transmission of some critical information. To this point, SPC counselors desired greater context for the referral, for example, more awareness of the patient’s level of interest vs. hesitancy towards the program which could help the counselor to tailor their approach when contacting the patient. Challenges with handoffs between agencies is likely a product of the structure of 24/7 referrals that are transmitted telephonically in voicemail rather than live telephonic contact between the psychiatry resident and SPC counselor. This mode of information transmission supports a 24/7 function and is efficient but may fail in providing enough data, especially given the complexity of many patients with suicidal ideation or behaviors. Efficiency of referrals could perhaps be supported by electronic messaging between agencies with more information on patient/family context. This could include mutually agreed upon data-sharing information that could help maximize SPC counselors’ ability to engage and assist patients with increased suicide risk following discharge.

Since SPC counselors had concerns about maintaining confidentiality when they were unable to reach a patient directly on a follow-up call, further work is needed to optimize balance between efficiency of making inter-agency referrals on a 24/7 basis, with the need for more detailed patient data and context. Hospitals or ED’s that operate their own caring contact program may have an advantage with greater ease of access to electronic health information, with the trade-offs of needing to train their own SPC counselors and manage the costs of the EFU program, an important issue for future research and quality improvement.

Nevertheless, there was a disconnect experienced by both agencies, concerning inadequate knowledge of the other, absence of feedback on referral outcomes, and feeling challenged about lack of a clean handoff between the ED and follow-up services. Enhanced technology for the program and deeper investment in the development of the structured partnership will require more rather than less effort in the future from both partners. Specifically, the desire for residents and SPC counselors to know each other could potentially be addressed by more regular recurring meetings or in-services between EFU program leadership and/or providers.

Partners on both sides of the EFU program lacked substantive feedback about program outcomes and effectiveness. SPC counselors had anecdotal evidence that patients appreciated their extra support while waiting for linkage to ongoing mental health care. However, psychiatry residents lacked any feedback, keeping them in the dark about services used and whether or not the EFU program improves patient outcomes. This lack of a feedback loop bred skepticism among residents, which led to the reluctance of referring patients into the program, and calls for efforts to provide detailed information to both partners. Innovative approaches to enhance communication are warranted such as collaborative quarterly virtual meetings, the making and sharing of videos about each site, or a website with photos and descriptions that is made for the staff of the ED and the program that provides needed details about each partner and the program process.

The 988 Suicide and Crisis Lifeline offers best practice guidelines for follow-up programs [15]. Recommendations include the use of easy reporting tools for electronic tracking of key program outcomes and user satisfaction surveys. However, the adoption of new inter-agency practices first requires one partner to establish proper communication channels, then to frame new practices in terms of benefits and risk reduction [1]. If appropriately adapted to the context of systems and patient needs, the implementation of an electronic reporting mechanism has the potential to improve program operations and therefore validate the need for continued services.

### Limitations

Qualitative data were from the perspective of providers involved in a partnered suicide EFU program for an academic institution and community outreach program. Therefore, results from the study are limited to this inter-agency partnership and not generalizable to other suicide prevention programs. Furthermore, we recruited a convenience sample of psychiatry residents and SPC counselors who were currently working in the ED and regional suicide center. Future exploration of the perspectives of other ED providers (supervising psychiatrists, nurse practitioners, physician assistants, registered nurses, and social workers), other suicide prevention program employees (supervisor or director), and patients/families could broaden understanding of program benefits and operations. Finally, patients discharged from the inpatient psychiatric setting were also offered follow-up services. Future investigation should explore perspectives of inpatient psychiatrists and other providers, since differences in EFU program referral practices, such as time available to prepare and refer patients, could vary greatly between ED and inpatient psychiatrists.

## Conclusion

Programs for reducing risk of suicide or self-harm following discharge from the emergency department are critically important. “Caring contacts” are brief but highly valuable communications with patients after discharge from the ED or hospital, and have been endorsed by multiple agencies, including AHRQ, SAMHSA, and the Suicide Lifeline. Partnerships between hospitals and local suicide prevention centers have many potential benefits for patients and the healthcare system alike, however these programs require careful study to assure that the people in need are effectively getting connected with follow-up care and not falling through the cracks, so that the system is operating as efficiently as possible. Careful analysis is also needed to document and appropriately justify the costs of such programs.

This study provides a qualitative appraisal of such a program. The results demonstrated that there are benefits to this community partnership that effectively provide a “caring contact” hand-off. Psychiatry residents in our sample appreciated how such referrals reduced the burden of patient responsibility and was an improvement above the typical standard of care of providing a list of referrals that otherwise require the patient to act upon and initiate their own follow-up. SPC counselors appreciated the ability to develop meaningful relationships with patients over an extended period of time and valued their role as a facilitator of transition of care from the ED discharge.

However, this study identified a number of important challenges and areas of opportunity for future work. The results highlight the need for improvement in the quality of information transmitted between the ED and the SPC, which would enable the counselors to approach the patient in a more sensitive and fine-tuned manner. Additionally, further study is needed regarding the potential for difference in patients with chronic and/or recurrent suicidal ideation compared to new-onset suicidal ideation. It remains unclear if the program benefits certain populations more than others, or if specialized training based on these differences would be beneficial.

While efficacy of workflows was highly valued by the ED team, including recommendations to develop automated transmission of the referral to the Suicide Prevention team through the electronic health record, such efforts would need to keep in mind that counselors valued more thorough patient information and clinical context. Both psychiatry residents and counselors desired to have a better connection or awareness of the other, as well as more formal feedback on the success or failure of the referral to the outreach team. This may further inform if the outreach should be internal or external to the ED system, another important future research effort.

Overall, this study provides qualitative evidence that this partnered program is valued as a whole. Improved communication, implementation, consistent provider training, efficient and accurate outcome reporting mechanisms, and coordination between partner systems would likely lead to a better provider and patient experience. Such a program is potentially sustainable, particularly with consistent funding for ongoing collaboration.

## Data Availability

Please contact Dr. Patricia Soderlund if someone wants to request data from our study. The datasets used and/or analyzed during the current study available from the corresponding author on reasonable request.

## Abbreviations

AHRQ: Agency for Healthcare Research and Quality
ED: Emergency Department
EHR: Electronic Health Record
EPIS: Exploration, Preparation, Implementation, and Sustainment
EFU Program: Extended Follow-Up Program
SAMHSA: Substance Abuse and Mental Health Services Agency
SPC: Suicide Prevention Center
SPRC: Suicide Prevention Resource Center
QI: Quality Improvement
